# 372. Co-Administration of OVX836, NP-Based Universal Influenza Vaccine Candidate, with Conventional HA-Based Influenza Vaccine: Results of Phase 2a Clinical trial

**DOI:** 10.1093/ofid/ofad500.442

**Published:** 2023-11-27

**Authors:** Paul Willems, Jessika Tourneur, Delphine Guyon-Gellin, Alexandre Le Vert, Florence Nicolas

**Affiliations:** Osivax, Lyon, Rhone-Alpes, France; Osivax, Lyon, Rhone-Alpes, France; Osivax, Lyon, Rhone-Alpes, France; Osivax, Lyon, Rhone-Alpes, France; Osivax, Lyon, Rhone-Alpes, France

## Abstract

**Background:**

Quadrivalent Inactivated Influenza Vaccine (QIV) generate antibody responses mostly directed against the highly mutating hemagglutinin. An alternative path for influenza vaccination is to generate cellular immunity to well-conserved nucleoprotein (NP), which has been associated with protection against influenza disease.

OVX836 is an unadjuvanted recombinant vaccine targeting NP. We have previously shown that OVX836 induces strong, dose-dependent NP-specific T and B-cell immune responses in human, with a signal for efficacy of 84% (95%CI=17%-97%), together with synergistic protection with QIV in preclinical models.

Here, we investigate in human the concomitant administration of OVX836 with QIV.

**Methods:**

Phase 2a, randomized, double-blind, controlled, study to evaluate immunogenicity and safety of the concomitant administration of OVX836 and QIV in healthy adults (18-55 years) as 2 separate injections into the same arm, compared to (i) QIV and (ii) OVX836.

**Results:**

Safety: All three treatments were safe and well tolerated. All occurrences of local and/or systemic signs and symptoms were mild or moderate in severity, except for one severe fatigue and myalgia in the QIV group and one severe headache in the OVX836 group.

Immunogenicity: Primary endpoint was achieved for the QIV and the OVX836+QIV, which triggered adequate immune response to the QIV (Hemagglutination Inhibition - HAI). Geometric mean fold-rise at Day 29 vs Day 1 of the HAI were similar between the QIV and OVX836+QIV groups for all strains contained in the QIV (figure 1). While there was an increase in NP-specific T-cell IFN-γ ELISPOT response at Day 8 for the OVX836 and the OVX836+QIV groups compared to QIV (p< 0.0001, Wilcoxon rank-sum test), no significant difference was observed in terms of change (D8-D1) between OVX836 and OVX836+QIV – figure 2.

The analysis of additional NP-specific immune parameters supports similar conclusion, with no to very limited immune interference between vaccines.

**Figure 1:**
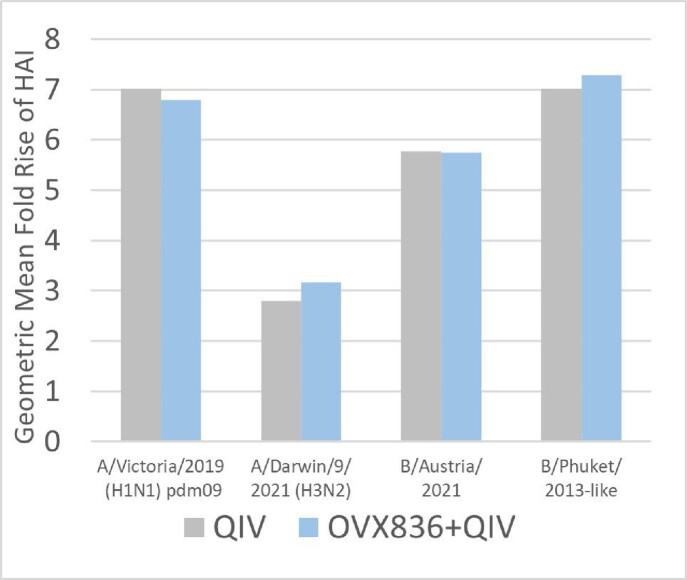
Geometric Mean Ratio of HAI for each strain of the QIV between D1 and D29

**Figure 2:**
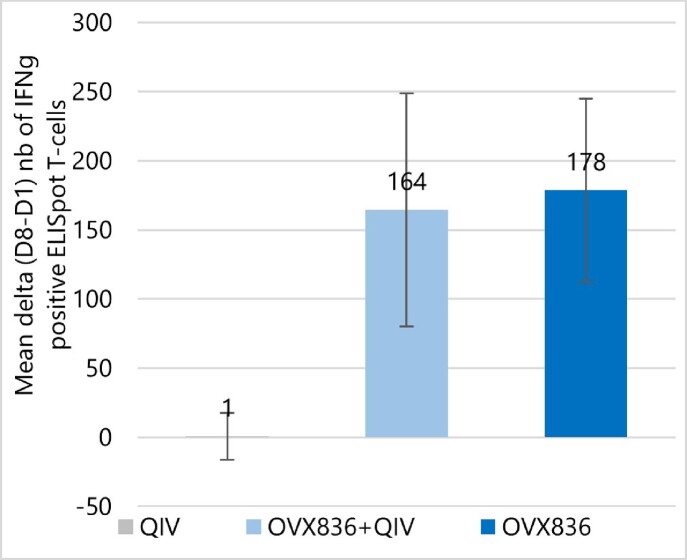
Mean Change (+Standard Deviation) of IFNg ELISpot response b/w D1 and D8

**Conclusion:**

OVX836 co-administered with QIV was safe, with no immune interference on either HAI or NP-specific IFN-γ ELISPOT, thus warranting further evaluation in larger trials.

**Disclosures:**

**Paul Willems, n/a**, Osivax: Advisor/Consultant **Jessika Tourneur, n/a**, Osivax: Stocks/Bonds **Delphine Guyon-Gellin, n/a**, Osivax: Stocks/Bonds **Alexandre Le Vert**, Osivax: Board Member|Osivax: Stocks/Bonds **Florence Nicolas, n/a**, Osivax: Stocks/Bonds

